# Histone deacetylase 10, a potential epigenetic target for therapy

**DOI:** 10.1042/BSR20210462

**Published:** 2021-06-04

**Authors:** Fajuan Cheng, Bin Zheng, Jianwei Wang, Guiting Zhao, Zhongshun Yao, Zhihong Niu, Wei He

**Affiliations:** 1 Department of Nephrology, Shandong Provincial Hospital Affiliated to Shandong University, Jinan, Shandong, P.R. China; 2 Department of Nephrology, Shandong Provincial Hospital Affiliated to Shandong First Medical University, Jinan, Shandong, P.R. China; 3 Cheeloo College of Medicine, Shandong University, Jinan, Shandong, P.R. China; 4 Department of Urology, Shandong Provincial Hospital Affiliated to Shandong First Medical University, Jinan, Shandong, P.R. China; 5 Department of Urology, Shandong Provincial Hospital Affiliated to Shandong University, Jinan, Shandong, P.R. China; 6 Department of Urology, Shandong Provincial ENT Hospital Affiliated to Shandong University, Jinan, Shandong, P.R. China

**Keywords:** cancer, disease, epigenetics, HDAC10, histone deacetylases

## Abstract

Histone deacetylase (HDAC) 10, a class II family, has been implicated in various tumors and non-tumor diseases, which makes the discovery of biological functions and novel inhibitors a fundamental endeavor. In cancers, HDAC10 plays crucial roles in regulating various cellular processes through its epigenetic functions or targeting some decisive molecular or signaling pathways. It also has potential clinical utility for targeting tumors and non-tumor diseases, such as renal cell carcinoma, prostate cancer, immunoglobulin A nephropathy (IgAN), intracerebral hemorrhage, human immunodeficiency virus (HIV) infection and schizophrenia. To date, relatively few studies have investigated HDAC10-specific inhibitors. Therefore, it is important to study the biological functions of HDAC10 for the future development of specific HDAC10 inhibitors. In this review, we analyzed the biological functions, mechanisms and inhibitors of HDAC10, which makes HDAC10 an appealing therapeutic target.

## Introduction

Nuclear DNA in eukaryotic cells always combines with histones before packaging into the chromatin. However, a fundamental question is how the chromatin is regulated when and where it is necessary. The existing evidence presented that ATP-dependent chromatin remodeling as well as covalent chromatin modification may contribute to this regulation [1]. Covalent chromatin modification includes acetylation, phosphorylation, methylation, ubiquitination and ADP-ribosylation [2,3]. Acetylation, as the first discovered modification, is the most studied and best characterized among all modifications. There is compelling evidence that acetylation of lysine residues at amino acid termini greatly affects transcriptional regulations [2].

Histone deacetylases (HDACs) serve as an ‘eraser’, which remove acetate from acetylated histone as well as other non-histone proteins [4,5]. Generally, HDACs are also known as lysine acetyltransferases as well as lysine deacetylases [6]. Additionally, HDACs exert effects on post-translational modifications, such as ubiquitination and methylation, and can influence gene transcription by increasing the interaction between DNA and histone [7–9]. In eukaryotic cells, HDACs may come from prokaryotic enzymes, similar to acetylpolyamine amidohydrolases [7,10]. Currently, HDACs are divided into four classes [11]. In 2002, Fischer et al. [12], Tong et al. [5], Kao et al. [13] and Guardiola and Yao [14] reported a novel enzyme that shares functional characterizations with other class II members, and named it HDAC10.

In this review, we focus our attention on the progress of studies on the role of HDAC10 in various tumors and non-tumor diseases. Particular emphasis is given to its functions and mechanisms.

## Structures and catalytic mechanisms of different HDACs

### Structures of class II HDACs

Class II HDACs are subclassified as IIa and IIb. Class IIa HDACs (HDAC4, 5, 7, 9) are defined by a functionally essential N-terminal domain, which can regulate nuclear–cytoplasmic shuttling as well as specific DNA-binding. The cellular trafficking of class IIa HDACs is determined by intrinsic nuclear import or export signals as well as specific binding sites for 14-3-3 proteins [15]. Through binding with the 14-3-3 proteins, Class IIa HDACs can stimulate cytoplasmatic retention and nuclear export in a phosphorylation-dependent manner. These processes in turn affect the activity of transcription factors, such as myocyte enhancing factor-2 [16,17].

Class IIb HDACs include HDAC6 and HDAC10. HDAC6 contains two tandem deacetylase domains and a C-terminal zinc finger [18]. HDAC10 is structurally related to HDAC6. The HDAC10 gene localizes to chromosome 22 [14]. Consisting of 20 exons, HDAC10 contains two spliced transcripts [5,12] and also contains N-terminal catalytic domain and C-terminal leucine-rich domain. Specifically, the N-terminal catalytic domain of HDAC10 is similar to the deacetylase domain of other known class II HDACs, but the C-terminal catalytic domain does not contain residues that are necessary for enzymatic functions. The existence of both domains may confer resistance to trapoxin B and sodium butyrate [5,14]. HDAC10 is member of the arginase/deacetylase superfamily and is expressed differentially in the cytoplasm and nucleus [10,12]. It may also repress transcription except for the deacetylation activity in the nucleus [14]. Moreover, HDAC10 expressed in most human tissues, including the kidney, pancreas, liver, spleen, heart, testis, brain and placenta [5]. Emerging evidence has established that HDAC10 is responsible for the regulation of polyamine. Using X-ray crystallography, researchers have observed that HDAC10 has a unique conserved glutamate gatekeeper that may promote N^8^-acetylspermidine hydrolysis, serving as a polyamine deacetylase (PDAC) [6,19].

Noticeably, class II HDACs show 23–81% amino acid similarity with conserved deacetylase domains. Moreover, they all have cytoplasmic localization, which implies that class II HDACs play significant role in cytoplasmic functions [13,20,21].

### Catalytic mechanisms of HDACs

Class I, II and IV HDACs require zinc ion to exert enzyme activity. The X-ray crystallography has determined catalytic domain structures of Class I, II and IV HDACs, which contain open α/β-family of folds and stranded parallel β-sheets [22]. These structures make contributions to the binding of HDACs active sites and HDAC inhibitors (HDACIs). A model of action proposed by Wu et al. [23] suggested that H143, a histidine residue, could function as a base to accept protons from zinc-bound molecules in a rate-determining step. Then the accepted proton will be shuttled to the amide nitrogen atom to facilitate the cleavage of amide bond [24].

The class III HDACs has a common model of action that requires cofactor NAD^+^ [25]. A structural study found that the catalytic domain of class III HDACs locates in a cleft, which could constitute a protein tunnel where the substrate could combine with NAD^+^. Mechanically, based on the catalytic structure of class III HDACs, cleavable nicotinamide from NAD^+^ and ADP-ribose could transfer to acetylated lysine [26].

## Biological characterization and mechanism of HDAC10 in tumors

**Figure 1:**
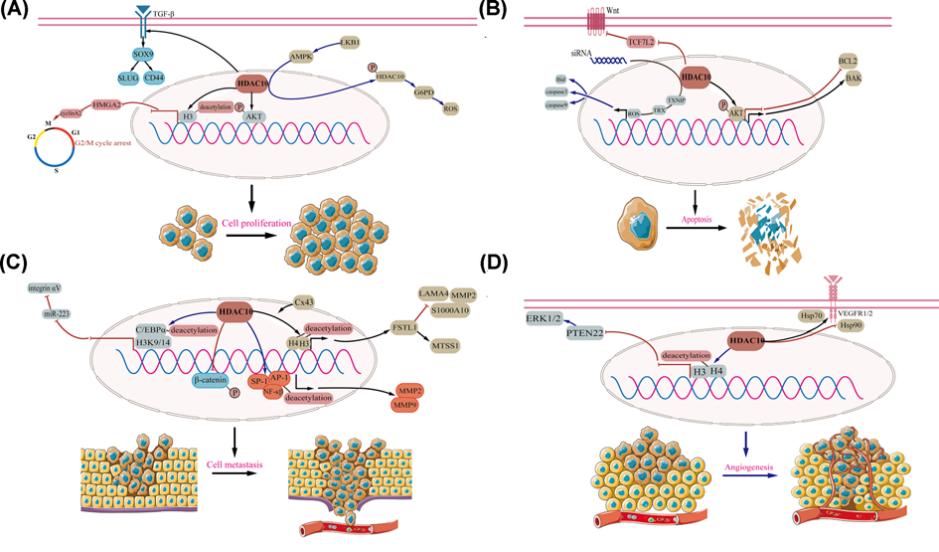
HDAC10 regulate cell proliferation, cell apoptosis, cell metastasis and angiogenesis in cancer cells (**A**) Roles of HDAC10 in cell proliferation. (**B**) Roles of HDAC10 in cell apoptosis. (**C**) Roles of HDAC10 in cell metastasis. (**D**) Roles of HDAC10 in angiogenesis.

In 2000 and 2011, Hanahan and Weinberg proposed six and additional four hallmarks of tumors, respectively [27,28]. These conceptions promoted the understanding of the diversity of tumors. Here, we focus mostly on HDACs that affect the biology of tumors and tumorigenesis, and summarize the mechanistic underpinnings concerning cell proliferation, cell apoptosis, cell invasion, migration, metastasis, angiogenesis and other tumor-related biological functions (Figure 1 and Table 1).

**Table 1:** Overview of biological roles of HDAC10 in several malignant tumors

Tumor	Expression level	Function	Target/Signaling pathway	Cell line/Tissue	Reference
NSCLC	Elevated	Cell proliferation, Cell apoptosis	AKT, BCL2, BAK	A549, H358 and H460 cell	[18]
Lung adenocarcinoma	Elevated	Cell proliferation, cancer stemness, Tumor immune	TGF-β pathway, SOX9, CD44, SLUG	HDAC10 KO mice, HDAC10 WT mice	[19]
Lung cancer	Elevated	Cell proliferation	LKB1–AMPK signaling, G6PD, ROS	H1299, H157, H1944, H460 and A549 cell	[23]
Lung cancer	Elevated	Cell proliferation, Cell cycle	H3, HMGA2	H1299, H441, H23, H157, H2122, H358, A549, PC9, H1975, H322, H292, H460, H522, H661 and ADLC-5M2 cell	[25]
Lung cancer	Decreased	Cell metastasis	Cx43, FSTL1, H3, H4, S100A10, MMP-2, LAMA4, MTSS1	PG cell	[41]
RCC	Decreased	Cell proliferation, cell invasion	β-catenin	ACHN, Caki-2 cell, 45 primary RCC tissues and adjacent normal tissues	[20]
Gastric cancer	/	Cell apoptosis	Caspase-3, Caspase-9, Bid, TXNIP, ROS	SNU-620 cell	[35]
Gastric cancer	/	Angiogenesis	Hsp70, VEGFR1, VEGFR2	SNU620 cell	[51]
Colorectal cancer	/	Cell apoptosis	Wnt pathway, TCF7L2	SW480, HCT116 cell	[32]
Colon cancer	/	Angiogenesis	Hsp70, VEGFR1, VEGFR2	HCT116 cell	[51]
Colon cancer	Elevated	Mismatch repair	MSH2, MLH1, MSH6	100 colon cancer tissues	[81]
Cervical cancer	/	Cell metastasis	MMP-2, MMP-9	HeLa-S3 cell, 60 cancer tissue and normal tissue	[42]
Cervical cancer	/	Autophagy	LAMP2A-positive lysosome	HeLa cell	[75]
Cervical cancer	/	Mismatch repair	MSH2	HeLa cell	[80]
Cervical cancer and Osteosarcoma	/	Drug resistance	hSSB1, p300	HeLa and U2OS cells	[62]
HCC	/	Cell metastasis	H3K9/14, C/EBPα, miR-223, integrin αV subunit	SMMC-7721 cell	[43]
HCC	/	HBV-infected HCC	Promoter polymorphism	1095 HBV infection patient tissues	[82]
Neuroblastoma	/	Drug resistance	Lysosomal exocytosis, DNA damage	SK-N-BE (2)-C, IMR-32 and SK-N-AS cell	[55]
Neuroblastoma	/	Drug resistance	Hsp70, Hsc70	BE [2]-C, Kelly and IMR32 cell	[57,58]
Ovarian	Decreased	Drug resistance	DNA repair	UWB1.289 cell	[59]
Prostate cancer	/	Drug resistance	AR-V7, flAR, BRD4, NCOR2, DUB3, BRD4	C4-2, PC-3, 22Rv1, DU145, LNCaP, VCaP and LAPC4 cells	[66,67]
Adrenocortical cancer	/	Chromatin modulator	Methylation	44 adrenocortical cancer and 6 normal tissues	[83]
Melanoma	Decreased	Gene expression	HDAC	Melanoma tissue and normal samples	[84]

Abbreviations: AKT, protein kinase B; AMPK, AMP-activated protein kinase; AR-V7, androgen receptor-V7; BAK, BCL2 antagonist/killer; BCL2, B-cell lymphoma-2; Bid, BH3 interacting domain death agonist; BRD4, bromodomain-containing protein 4; Cx43, connexin 43; C/EBPα, CCAAT enhancer-binding protein; DUB3, ubiquitin-specific peptidase 17 like family member 2; flAR, full length androgen receptor; FSTL1, follistatin-like 1; G6PD, glucose-6-phosphate dehydrogenase; HBV, hepatitis B virus; HCC, hepatic cell carcinoma; HMGA2, high mobility group A 2; Hsp70, heat shock protein 70; hSSB1, human single-stranded DNA binding protein; LAMA4, laminin subunit α 4; LAMP2A, lysosomal associated protein 2 A; LKB1, liver kinase B1; MLH1, mutL homolog 1; MMP-2, matrix metalloproteinase-2; MMP-9, matrix metalloproteinase-9; MSH2, mutS homolog 2; MSH6, mutS homolog 6; MTSS1, MTSS I-BAR domain containing 1; NCOR2, nuclear receptor co-repressor 2; NSCLC, non-small-cell lung cancer; RCC, renal cell carcinoma; ROS, reactive oxygen species; SOX9, sex-determining region Y box protein 9; TCF7L2, transcription factor 7 like 2; TGF-β, transforming growth factor-β; TXNIP, thioredoxin interacting protein; VEGFR1, vascular endothelial growth factor receptor 1; VEGFR2, vascular endothelial growth factor receptor 2.

### HDAC10 and cell proliferation

HDAC10 plays an essential role in the regulation of cancer cell growth. It has been demonstrated that it can prompt cell proliferation in non-small-cell lung cancer (NSCLC) [29]. In contrast, HDAC10 suppresses cancer cell growth in other malignant tumors, including lung adenocarcinoma [30] and renal cell carcinoma (RCC) [31]. Akt is a key molecule in the PI3K-AKT pathway, and is involved in cell proliferation, survival and other key functions [32]. Analogously, HDAC10 overexpression significantly facilitates NSCLC cell growth by regulating the Ser^473^ phosphorylation of AKT [29]. In lung adenocarcinoma, deletion of HDAC10 accelerates the progression of KRAS-driven cancer both *in vivo* and *in vitro.* Specifically, by activating the transforming growth-factor β (TGF-β) pathway, deletion of HDAC10 promotes the expression of sex-determining region Y box protein 9 (SOX9), which subsequently up-regulates the expression of SLUG, as well as CD44. These processes contributes to the growth of lung cancer spheres via SOX9-mediated stem-like properties, suggesting that HDAC10-TGF-β-SOX9-SLUG/CD44 axis plays an essential role in lung adenocarcinoma [30].

AMP-activated protein kinase (AMPK) regulates biological functions in tumors that are mediated by liver kinase B1 (LKB1), such as cell survival and transcription, via the mTOR pathway [33]. Induced by LKB1–AMPK signaling, phosphorylated HDAC10 is transported from the the nucleus to the cytoplasm and further enhances the expression of glucose-6-phosphate dehydrogenase (G6PD). This decreases the level of reactive oxygen species (ROS) and promotes lung cancer cell proliferation [34]. The cases described above suggest that the LKB1–AMPK-HDAC10-G6PD-ROS pathway might be important to tumor cell proliferation. However, in RCC cells, suppressed expression of HDAC10 significantly promotes the phosphorylation of β-catenin and thus plays a part in anti-proliferation [31] (Figure 1A).

Perturbed cell cycle is also a growth-regulation way in cancer cell [35]. Previous studies have shown that by inhibiting histone H3 deacetylation around the let-7f-2/miR-98 promoter, HDAC10 suppresses HMGA2 expression which target to cyclin A2 promoter, and further inhibits the transcription of cyclin A2 [30,36]. The signaling pathways: HDAC10-let-7f-2/miR-98-HMGA2-cyclin A2 arrests the G_2_/M transition and finally inhibits lung cancer cell proliferation. Evidence suggests that both cell cycle inhibitors (such as P21 and P27) and promoters (such as cyclins E1 and D1) play a vital role in cancer progression [37–39]. HDAC10 plays an oncogenic role by inhibiting the expression of P27, P21 and enhancing that of cyclins D1 and E1 [29] (Figure 1A).

### HDAC10 and cell apoptosis

Cancer cells will not die in a scenario that too little apoptosis happens [40]. Numbers of proteins and signaling are involved in cell apoptosis. It has been established that overexpressed anti-apoptotic proteins (such as those in the Bcl-2 family) as well as down-regulated proteins (such as Bid, BIK and BAK) may disrupt the balance between apoptosis and anti-apoptosis [41,42]. By targeting AKT, HDAC10 affects the expression of B-cell lymphoma-2 (BCL2) as well as BCL2 antagonist/killer (BAK), which induces apoptosis in lung carcinoma [29]. In colorectal cancer, inhibited HDAC10 expression promotes cell apoptosis by depleting transcription factor 7 like 2 (TCF7L2), which attenuates the Wnt pathway [43]. ROS, generated from mitochondrial damage or oxidative stressors, may promote caspases and induce apoptosis [44,45]. Lee et al. found that a low level of HDAC10 in gastric cancer may activate proapoptotic molecules including caspase-3, caspase-9, and Bid through the thioredoxin interacting protein (TXNIP)-induced ROS signaling pathway [46] (Figure 1B). Although previous studies have determined limited mechanisms in lung, colorectal and gastric tumors, including the HDAC10-AKT-BCL2-BAK pathway, the HDAC10-TCF7L2-Wnt pathway and the HDAC10-TXNIP-ROS-caspase-3/caspase-9/Bid pathway, it is not yet clear whether these mechanisms exist in other tumors [29,43,46]. Therefore, advances in research on the mechanisms of HDAC10 will be key to unraveling its potential importance in tumors.

### HDAC10 and cell metastasis

Tumor cell metastasis is a multistep process including cell adhesion, invasion, migration and dissemination at distant organs [47,48]. Invasion- and migration-related molecules, such as matrix metalloproteinases (MMPs), and S100A10 are dysregulated in various cancer cell lines [49–51]. Zhao et al. [52] reported that HDAC10 shows low expression levels in pulmonary giant cell carcinoma cells and is subject to regulation by connexin 43 (Cx43). As Cx43 is overexpressed, the expression of follistatin-like 1 (FSTL1) is elevated, via the enhanced binding between HDAC10-mediated acetylation of H3 and H4 and the promoter of FSTL1. The Cx43-HDAC10-FSTL1 axis not only contributes to the low expression levels of S100A10, MMP-2 and laminin subunit α 4 (LAMA4), but also enhances the expression of MTSS I-BAR domain containing 1 (MTSS1), which plays a pivotal role in the suppression of both invasion and metastasis [52]. In metastatic cervical squamous cancer cells, HDAC10 serves a tumor suppressor role through the regulation of MMPs. Mechanistically, due to the combination of HDAC10 and the promoters of MMP-2 (especially the AP1-binding site) and MMP-9 (especially the NF-κB- and sp1-binding sites) the decreased level of histone acetylation impedes the binding function of polymerase II, weakens the expression levels of MMP-2 and MMP-9 and restrains cancer cell invasion and migration [53]. HDAC10 also deacetylates acetyl-H3K9/14 and acetyl-C/EBPα, preventing their recruitment to the promoter of miR-223 in sulfatide-treated hepatocellular cancer (HCC) cells. The low expression of miR-223 further facilitates the expression of integrin αV subunit and then attenuates HCC cell migration as well as metastasis [54]. The β-catenin pathway is altered in various tumors and also plays important roles in carcinogenesis [55,56]. In renal cell carcinoma (RCC), overexpressed HDAC10 restrains RCC cell invasion by inhibiting phosphorylated nuclear β-catenin expression [31] (Figure 1C). Although these studies are interesting, they were mainly based on overexpression experiments. Therefore, the mechanism of HDAC10 in tumor metastasis must be characterized in more detail.

### HDAC10 and angiogenesis

Angiogenesis is an indispensable process in the early stage of tumor growth [57]. Angiogenesis involves several pro-angiogenic proteins, including vascular endothelial growth factor (VEGF), TGF-β, extracellular regulated protein kinases (ERKs) and others [58–60]. Duan et al. [61] reported that overexpressed HDAC10 deacetylates H3 and H4 in promoter of phosphatase and tensin homolog 22 (PTEN22) and consequently inhibits polymerase II from binding to PTEN22 promoter. The suppressed expression of PTEN22 subsequently increases ERK1/2 activation and ultimately facilitates the formation of tubes in *vivo* and in *vitro*. Because of this, it was speculated that the HDAC10-PTEN22-ERK1/2 signaling pathway may contribute to tumor angiogenesis. HDAC10 also regulates angiogenesis via depletion of VEGFR in gastric and colon cancer cells. Mechanistically, HDAC10 obstructs heat shock protein (Hsp) 90 bound to VEGF receptor 1 (VEGFR1)/VEGFR2, but promotes the combination between Hsp70 and VEGFR1/VEGFR2 [62]. This imbalanced binding leads to proteasomal-dependent degradation of VEGFRs, which may regulate angiogenesis (Figure 1D).

### HDAC10 and drug resistance

Drug resistance is a common phenomenon during chemotherapy. It not only limits the therapeutic effect but can even lead to treatment failure [63]. The altered drug metabolism, mutated drug targets and elevated drug efflux rate contribute to drug resistance [64,65]. Down-regulation of HDAC10 may restore the sensitivity of doxorubicin in neuroblastoma. Ridinger et al. [66] reported that suppressing HDAC10 expression promotes intracellular accumulation of doxorubicin by inhibiting lysosomal exocytosis, which results in cell death with enhanced double-strand breaks (DSBs) as well as DNA damage. Additionally, autophagy, activated via therapeutic stress, participates in drug resistance and multidrug resistance (MDR) [67]. Consistent with this point, Oehme et al. [68,69] found that HDAC10 deletion acetylated Hsp70/heat shock cognate 70 (Hsc70), and impairs the autophagic flux by thwarting the blend between autophagosomes and lysosomes, which allows drug-resistant neuroblastoma cell to recover sensitivity to doxorubicin. A study that combined bioinformatics analyses and cell experiments found that low HDAC10 expression both intensifies the cytotoxicity of ovarian cancer cells to cisplatin and constrains DNA repair [70].

Many cytotoxic events can break DNA double strands and result in cell death [71]. Encountering DNA damage, various proteins, such as human single-stranded DNA binding protein 1 (hSSB1), will repair the flawed sites [72]. Wu et al. [73] found that p300 acetylates hSSB1 Lys^94^ when it localizes to impaired sites, and recovers DNA stability. However, up-regulated HDAC10 reverses this process and makes tumor cells more responsive to chemotherapy. Castration-resistant prostate cancer (CRPC), arising from resistance to androgen deprivation therapy (ADT), is a tricky problem [74]. Previous studies have reported that bromodomain and extra-terminal protein inhibitor (BETi) can deteriorate the androgen receptor (AR) signaling pathway. Moreover, in prostate cancer cells, elevated expression of bromodomain-containing protein 4 (BRD4, a member of BET family), induced by BETi, confers resistance to these inhibitors [75,76]. Jin et al. [77] found that low expressed nuclear receptor co-repressor 2 (NCOR2)–HDAC10 complex is negatively associated with deubiquitinase ubiquitin-specific peptidase 17 like family member 2 (DUB3), which may interact with BRD4 and further inhibit sensitivity to BETi in prostate cancer cells and *in vitro*. Although the roles of HDAC10 in drug resistance have not been explored completely, experiments suggest that the NCOR2-HDAC10-DUB3-BRD4 signaling pathway might be useful as a regulator of drug resistance in tumors [77]. AR is a significant target in treating CRPC, and silencing HDAC10 also reduces the transcriptional activity of AR-V7 and full-length AR (flAR) in prostate cells [78], which implies that HDAC10 has potential therapeutic value.

### Other biological functions

Cancer stem cells (CSCs) are cells hidden in tumors that are capable of promoting tumor growth, progression and resistance treatments [79,80]. Many molecules, such as SOX9, SLUG and CD44, may help determine stem cell state [81,82]. In lung adenocarcinoma, SOX9, SLUG and CD44 are activated by the down-regulation of HDAC10 and stimulation of TGF-β, which mediates CSC-related cancer progression [30]. HDAC10 impacts immune and inflammation function as well. HDAC10-deleted Foxp3^+^ Treg cells have immunosuppressive functions and may play roles in alleviating immune-related colitis and prolonging cardiac allograft survival in MHC-mismatched mice [83]. HDAC10 deletion also recruits massive macrophages (especially M2 macrophages) into the lung cancer microenvironment. Considering that M2 macrophages may secrete anti-inflammatory molecules and promote tumor activity, HDAC10 may accelerate tumor progression by increasing tumor inflammation [30,84]. An additional effect of deletion of HDAC10 is chaperone-mediated autophagy (CMA), a form of autophagy that occurs via selective cytosolic protein transfer [85]. In knockout HDAC10 cervical cancer cells, Obayashi et al. [86] identified that increased expression levels of lysosomal associated protein 2 A (LAMP2A) and increased accumulation of LAMP2A-positive lysosomes near the nucleus raised, which may be a sign of activated CMA [87,88]. This characteristic may be effective for cancer treatment.

The mismatch repair system (MMR) is important in correcting flaws occurring during the stage of DNA replication [89]. It has been shown that mutL homolog 1 (MLH1), mutS homolog 2 (MSH2) and MSH6 commonly participated in MMR [90]. HDAC10 can trigger MSH2 activity initiated by MSH2 deacetylation at Lys^73^ in HeLa cells, following improved MMR activation [91]. A study that compared colon cancer cells and adjacent cells also revealed that low HDAC expression level in normal cells were negatively correlated with MSH2, MLH1 and MSH6 [92]. This may account for the different prognoses between normal and tumor cells.

**Figure 2:**
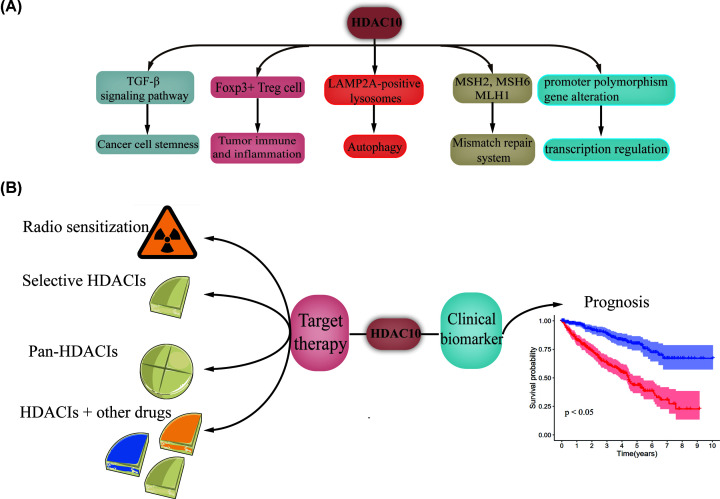
Role of HDAC10 in non-tumor diseases and its clinical applications (**A**) Role of HDAC10 in non-tumor diseases. (**B**) Clinical applications of HDAC10.

HDAC10 can perform regulatory roles through other epigenetic modifications, i.e. transcription regulation, methylation and promoter polymorphism [93–95]. In adrenocortical cancer cells and adenoma cells, HDAC10 is significantly methylated and its expression level dramatically decreases with hypermethylation [94]. Uzdensky et al. [95] observed down-regulated HDAC10 during transcription deregulation of melanoma progression. HDAC10 also participates in influencing the occurrence and onset age of HBV infection in HCC patients due to the promoter polymorphism, notably HDAC10-589C>*T*. The ‘T’ allele in HDAC10-589C>*T* enhances transcription activity, and might spur HCC progression by increasing HDAC10 expression [93]. Other study identified that frameshift deletions, one type of repeated alterations, occurred in HDAC10 among anaplastic thyroid cancer specimen [96]. According to Lourdusamy et al. [97], a large number of genes, including *BRD1*, *HIRA* and *HDAC10* are expressed at low levels in spinal ependymoma. However, the detailed mechanisms underlying the activity of HDAC10 in spinal ependymoma still need to be elucidated. These studies may manifest an alternate form of tumor regulation (Figure 2A). It is worth noting that the specific mechanisms of the involvement of HDAC10 in other functions also remain unresolved, and further studies are needed. Nonetheless, the studies reviewed herein demonstrate the promise of HDAC10 as a molecular target for cancer.

## Prognostic roles of HDAC10

The potential prognostic roles of HDAC10 have been explored. Up-regulated HDAC10 is associated with favorable overall survival (OS) in colon cancer, gastric cancer, RCC and NSCLC patients [31,92,98,99]. However, it should be noted that Liu et al. [100] studied the OS data of 180 NSCLC patients and found that up-regulated HDAC10 predicted poor OS. Moreover, HDAC10 is also an independent predictor of treatment-free survival (TFS) as well. HDAC10 level is associated with poor TFS in chronic lymphocytic leukemia (CLL) [101]. In this disease, high expression levels of HDAC are indirectly associated with a poor prognosis [102–104]. PD-L1 is expressed in various cancers and its clinical therapeutic value has been validated [105]. Due to enhanced PD-L1 expression in NSCLC patients with high HDAC10 expression, HDAC10 could function as a biomarker for the evaluation of PD-L1 treatment [100]. The elevated radiosensitization caused by inhibiting HDAC10 involves down-regulation of Rad51a, a component of DNA repair pathways [106]. In addition, positive HDAC10 expression is correlated with adverse clinical features, such as larger tumor size, poor tumor stage and more lymph node metastasis [99] (Figure 2B).

## HDAC10 in other non-tumor diseases and its pathophysiological functions

### Respiratory system diseases

An important role of HDAC10 has been found in asthma-induced eosinophilic airway inflammation. Zhang et al. [107] demonstrated that glucocorticoid (GC) and anacardic acid (AA) have the ability to alleviate IL-4, IL-5 and IL-13 and ameliorate airway inflammation *in* *vivo*, which depends on the low histone acetylation induced by elevated HDAC10 in memory T cells. A study that examined titanium dioxide (TiO_2_), a latent occupational carcinogen [108] in lung cells, reported that TiO_2_ particles provoked ROS (especially superoxide) and HDAC10 expression after lung cell are treated at 100 and 160 μg/cm^2^ for 24 h, respectively [109]. This implies that heritable epigenetic changes may serve as a reply to TiO_2_ particle exposure. Moreover, HDAC10 is also a potential pathogenic gene in emphysema [110].

### Urinary system diseases

HDAC10 is also a latent biomarker in immunoglobulin A nephropathy (IgAN) and renal fibrosis as well [111,112]. HDAC10 protein levels are significantly higher in fibrotic kidneys. However, piceatannol treatment does not diminish HDAC10 expression but the results hint that HDAC10 may regulate renal fibrosis via other signaling pathways [111]. To determine a biomarker for IgAN, Guo et al. [112] utilized samples from the Gene Expression Omnibus (GEO) database and found that HDAC10 existed in monocytes of IgAN samples, which enriches in both alcoholism and chromatin modifying enzymes via construction of functional interaction (FI) network.

### Nervous system diseases

HDAC10 is associated with many nervous system diseases. Kebir et al. [113] indicated that one single nucleotide polymorphism (SNP) (rs7634112) located in HDAC10 is significantly associated with schizophrenia. They devised a model that incorporates HDAC10, HDAC3 and HDAC9 to divide schizophrenia patients with high accuracy. A Korean study confirmed these results and selected a new SNP (rs1555048) for the Korean population [114]. Wang et al. [115] reported that decreased HDAC10 expression may aggravate post-intracerebral hemorrhage inflammation by elevating downstream PTPN22 and activating NLRP3 inflammasomes in rats. HDAC10 can modulate drug addiction as well. González et al. [116] reported that single-dose methamphetamine (METH) enhances HDAC10 expression in the medial prefrontal cortex of rat and that such up-regulation influences H4ac enrichment, which may account for the modified cognitive functions observed after using psychostimulants. The translocation of Olig1 protein involves brain development as well as multiple sclerosis [117,118]. In oligodendrocytes, HDAC10 represses Olig1 Lys^150^ acetylation, which leads to Olig1 protein being retained in cytoplasm [119]. In mesial temporal lobe epilepsy with hippocampal sclerosis (MTLE-HS), HDAC10 mRNA is significantly up-regulated. Meanwhile, increased HDAC10 expression has also been found in cytoplasm instead of the nucleus [120]. This subcellular distribution hints at potential role of HDAC10 in regulating MTLE-HS pathophysiology.

### Hormone regulation

Many studies have opened new doors into the potential functions of HDAC10 in hormone regulation. Xu et al. [121] found that the alteration of decreased luteinizing hormone (LH) surge stimulated by estradiol (E2) in middle age female rats may mediated by HDAC10. Specifically, in the anterior hypothalamus, the attenuated HDAC10 expression and enhanced H3 acetylation induced by E2 were not observed in middle-aged female rats. A study from China illustrated the important role of HDAC10 in melanogenesis [122]. HDAC10 restores melanogenesis by deacetylating paired box protein 3 (Pax3) and KRAB-associated protein 1 (KAP1), which relieves the repressed promoters of microphthalmia-associated transcription factor (MITF) as well as tyrosinase-related protein 1 (TRP-1) and TRP-2 in melanoma cells [122]. These processes ultimately prove that melanogenesis is mainly achieved through HDAC10/Pax3/KAP1 pathway.

### Lipid metabolism

The role of HDAC10 in regulating lipid metabolism has also been investigated. Qian et al. [123] found that HDAC10 expression decreases in obese humans and other animals (including mice and monkeys). In one study, the level of HDAC10 was markedly reduced in the medial hypothalamus of fasting mice, but researchers did not investigate its impact on AcH3 and AcH4 expression [124]. A study of benzyl butyl phthalate (BBP) found that it reduces HDACs (including HDAC10) and further contributes to acetylation of H3K9, which drives adipogenesis in mesenchymal stem cells (MSCs) [125].

### Human immunodeficiency virus infection

The pathogenetic mechanisms of human immunodeficiency virus (HIV) are still being explored, which is crucial to develop blockbuster drugs [126]. Recent research suggests that HDAC10 takes part in HIV infectivity and thus can be used for its treatment [127–129]. Ran et al. identified that a virus-associated envelope glycoprotein (vEnv), one type of defective viral particle, mediates histone deacetylation via down-regulation of HDAC10 in Jurkat T cell, which ultimately elevates the infectivity of the virus and leads to HIV infection [127]. In CD4^+^ T cells, down-regulating HDAC10 expression promotes HIV-1 replication by increasing its interaction with DNA. Meanwhile, HDAC10 also combines with HIV-1 integrase (IN), lessening its reciprocal action with cellular protein lens epithelium-derived growth factor (LEDGF/p75), and ultimately deteriorating HIV-1 integration, which makes HDAC10 an inhibitor of HIV-1 replication [128]. Currently, antiretroviral drugs can prolong the life expectancy of HIV patients since it was introducted [130]. Rodriguez et al. reported that both raltegravir alone and in combination with morphine facilitate the level of HDAC10 [129]. However, further investigation is necessary to illustrate the underlying therapeutic mechanism.

### Other diseases

HDAC10 orchestrates various biological functions. Pinto et al. [131] found that HDAC10 may mediate nutrition-associated growth. After restricting food *in vivo* or starving serum *in vitro*, HDAC10 expression apparently increased which is induced by inhibiting mTOR, and elevated levels of HDAC10 activated autophagy via Hsp70 deacetylation, thereby repressing cell viability [131]. HDAC10 also affects inflammation in the temporomandibular joint. Down-regulated HDAC10 suppresses the IL-1β/NF-κB pathway and its related inflammatory response *in* *vitro* [132]. In addition, in embryonic as well as induced pluripotent stem cells, HDAC10 enhances the level of chromatin remodelers [133]. In retinal ganglion cells, excitotoxicity alters HDAC10 localization to the cytoplasm [134]. In patients with vasospastic angina, apparently DNA gain was observed in HDAC10 [135]. However, it remains to be explored the detailed HDAC10-related mechanism or therapeutic value (Table 2).

**Table 2:** Overview of selected biological roles of HDAC10 in non-tumor diseases

Disease	Expression level	Functions	Targets/Signaling pathways	Cell lines/Tissues/Animal model	Reference
Eosinophilic airway inflammation	Elevated	Inhibit airway inflammation	IL-4, IL-5, IL-13	CD4^+^ CD45RB^low^ cell	[96]
TiO_2_-associated lung disease	Elevated	Cellular response	Reactive oxygen species	A549 cell	[97]
Emphysema	/	Pathogenic gene	Copy number gains	32 emphysema blood samples	[99]
IgAN	/	Chromatin modifying enzymes	/	8 IgAN blood samples and 9 normal blood samples	[101]
Renal fibrosis	Elevated	Regulate renal fibrosis	/	C57BL/6 male mice	[100]
Schizophrenia	/	Diagnostic value	/	278 Korean schizophrenia patients, 626 Caucasian schizophrenia parents	[102,103]
Post-ICH inflammation	Decreased	Aggravate inflammation response	PTPN22/NLRP3	6 ICH samples and S-D rat	[104]
Drug addiction	Elevated	Affect cognitive function	H4ac enrichment	C57BL/6 mice	[105]
Multiple sclerosis	Decreased	Affect oligodendrocytes	Oligo1 lysine 150 acetylation	Mouse neural progenitor cells, HEK293T cell	[108]
MTLE-HS	Elevated	Transcription regulation	/	28 MTLE-HS samples	[109]
LH secretion	Decreased	Reduce LH secretion	H3 acetylation	S-D rat	[110]
Melanogenesis	/	Promote melanogenesis	Pax3/KAP1	HEK293 cell and B16F10 cell	[111]
Adipogenesis	Decreased	Promote adipogenesis	H3K9 acetylation	MSCs	[114]
HIV infection	Decreased	Promote HIV infection	IN/LEDGF/p75, histone deacetylation	CD4^+^ T cell	[116,117]
Nutrition-associated growth	Elevated	Inhibit cell viability	mTOR, Hsp70, autophagy	S-D rat, Huh7 cell	[120]
TMJ inflammation	Decreased	Inhibit inflammatory response	IL-1β/NF-κB	13 TMJ samples	[121]

Abbreviations: ICH, intracerebral hemorrhage; IL, interleukin; IN, HIV-1 integrase; mTOR, mammalian target of rapamycin; NF-κB, nuclear factor κB; NLRP3, NOD-like receptors 3; PTPN22, protein tyrosine phosphatase nonreceptor type 22; TMJ, temporomandibular joint.

## HDAC10 inhibitors

After constructing the homology model structure of HDAC10, Uba et al. further validated its reliability [136,137]. A group, focused on the tunnel behavior of HDAC10, suggested that the amino acids in tunnel-like sites of HDAC10 are subtly different from other HDACs (such as HDAC8 and HDAC11) identified a non-conserved amino acid in the wall of these sites [138]. Researchers have also found that HDAC10 contains Zn^2+^-binding groups and has additional interactions with inhibitors when accommodating with them [139]. Moreover, the X-ray crystal structure of zebrafish HDAC10 indicates that the gatekeeper E274 and humanized PEACE motif helix are the structural bases of highly selective HDAC10 inhibitors [140]. These results conduct a useful model and support the development of HDACIs with reliable information.

### Selective and non-selective HDAC10 inhibitors

**Figure 3:**
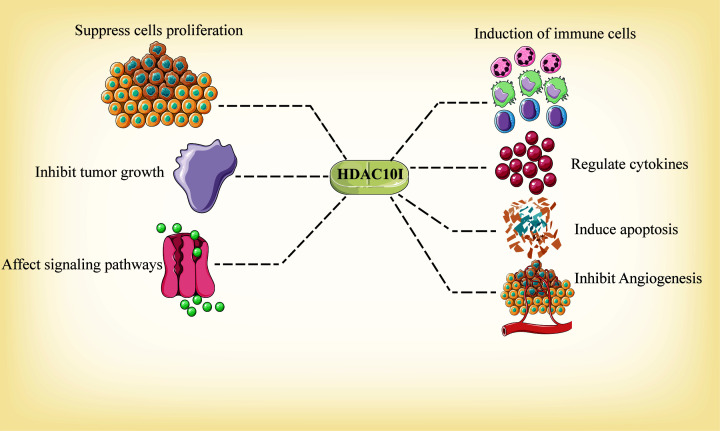
Mechanisms of action of selective and non-selective HDAC10 inhibitors

Nowadays, many HDACIs exhibit potent broad-spectrum repression ability to HDACs (including HDAC10), examples in broad-spectrum HDACIs include suberoylanilide hydroxamic acid [141], CRA-024781 [142], CRA-026440 [143], AR42 [144], trichostatin A (TSA) [145], valproic acid (VPA) [146] and so on. Both CRA-024781 and CRA-026440 are novel hydroxamic acid-based HDACIs that show potency against a series of HDACs, including HDAC1, 2, 3, 6, 8 and 10. CRA-024781 and CRA-026440 have *in vitro* efficacy in suppressing tumor cell proliferation as well as angiogenesis, inducing apoptosis and inhibiting tumor growth *in vivo* [142,143]. Another group reported pan-HDACIs AR42 and sodium valproate, which also have great selectivity over HDAC1, 3, 8 and 10 and obvious anti-tumor properties were observed *in* *vivo* and *in* *vitro* following pre-treatment with these HDACIs. Notably, AR42 and sodium valproate diminish PD-L1 and PD-L2 expression, and consequently elevate the efficacy of pazopanib, which increases multiple immune cells, for instance M1 macrophage, neutrophils, NK cells and T cells [144]. In addition, TSA can inhibit HDAC10 and 6, and the selectivity over HDAC10 arises from its N-terminal domain [147]. In colorectal cancer cells, TSA induces the inhibition of HDAC10 as well as HDAC6, and subsequently attenuates the Wnt pathway by the deletion of the Wnt signaling pathway-associated factor TCF7L2 in a proteasome-dependent way, which suppresses cell proliferation *in vitro* [43]. Bufexamac, an anti-inflammatory drug screened out via a new protocol, is also selective for HDAC10 and HDAC6 and its cellular potency may rely on interferon-α secreted by mononuclear cells [148] (Figure 3).

However, due to some severe adverse effects of multitarget HDACIs [149], Géraldy et al. [150] investigated HDAC10-specific inhibitors and found that tubastatin A tightly bound to HDAC10 in HeLa cells with its basic amine in the cap group. Based on an HDAC10 homology model, they revealed that a hydrogen bond falls in between a cap group nitrogen, and a gatekeeper residue Glu^272^ contributes to the tight binding. Unfortunately, however, the development of a highly selective HDAC10 inhibitor is still far off because of the inability to abrogate HDAC6 activity.

### Design or synthesis of HDAC10 inhibitors

We also examined previous works on the design or synthesis of HDAC10 inhibitors. By changing the condition of building block reaction, Villadsen et al. [151] synthesized azumamides A–E. In addition, they examined the inhibitory properties of HDAC10 under different drug concentrations (5 and 50 μM), which indicates both azumamides C and azumamides E markedly inhibit HDAC10. The HDACIs tubastatin A and PCI-34051 have the same N-benzylindole core [150,152]. After hybridizing these two inhibitors, Morgen et al. [153] synthesized dihydroxamic acids, which significantly suppressed the viability of neuroblastoma cells and played the role of potent inhibitor of HDAC10. Uba et al. [137] constructed M0017, the best model of human HDAC10, and filtered out ZINC19749069, the highest rank compound from the ZINC database, which displayed high stability when docking it into the catalytic channel of an HDAC10 model with quisinostat. Thus, M0017 and ZINC19749069 present considerable potential as an optimal HDACI and scaffold.

Although more and more studies could be reliably used for the HDACI designation, further exploration of the HDAC molecule structure and its biological characterization remain fundamental to shed light on the selection and development of the site-specific HDACIs.

## Conclusion

Like other HDAC isoforms, HDAC10 exhibits various complex functions and clinic values [4,154,155]. As mentioned above, its functions involve in DNA damage repair, gene transcription and autophagy, and HDAC10-mediated tumor cell proliferation, apoptosis, invasion, migration, angiogenesis, metastasis and immune regulation, laying the theoretical foundation for clinical application. However, there are still challenges to be met. First, more details on the mechanisms of aberrant HDAC10 regulatory are essential to provide a more integrative understanding of HDAC10 and other HDACs. Besides, it is yet to be determined how to treat patients with HDACI resistance as well as the precise dose of HDACI. Furthermore, it remains unclear whether extending HDACI to other solid tumors would lead to satisfactory outcomes. With further in-depth study, HDAC10 may serve as an essential anti-tumor target and contribute to clinical applications in the future.

## Data Availability

The present study includes no data deposited in external repositories.

## Abbreviations

AMPK: AMP-activated protein kinase
AR: androgen receptor
BAK: BCL2 antagonist/killer
BCL2: B-cell lymphoma-2
BETi: bromodomain and extra-terminal protein inhibitor
BRD4: bromodomain-containing protein 4
CMA: chaperone-mediated autophagy
CRPC: castration-resistant prostate cancer
CSC: cancer stem cell
Cx43: connexin 43
DUB3: deubiquitinase ubiquitin-specific peptidase 17 like family member 2
ERK: extracellular regulated protein kinase
E2: estradiol
FSTL1: follistatin-like 1
G6PD: glucose-6-phosphate dehydrogenase
HCC: hepatocellular cancer
HDAC: histone deacetylase
HDACI: HDAC inhibitor
HIV: human immunodeficiency virus
Hsp: heat shock protein
hSSB1: human single-stranded DNA binding protein 1
IgAN: immunoglobulin A nephropathy
KAP1: KRAB-associated protein 1
KRAB: kruppel-associated box domain
LH: luteinizing hormone
LKB1: liver kinase B1
MLH1: mutL homolog 1
MMP: matrix metalloproteinase
MMR: mismatch repair system
MSH2: mutS homolog 2
MTLE-HS: mesial temporal lobe epilepsy with hippocampal sclerosis
NCOR2: nuclear receptor co-repressor 2
NSCLC: non-small-cell lung cancer
OS: overall survival
Pax3: paired box protein 3
PD-L1: programmed cell death 1 ligand 1
PD-L2: programmed cell death 1 ligand 2
PTEN22: phosphatase and tensin homolog 22
SNP: single nucleotide polymorphism
SOX9: sex-determining region Y box protein 9
TCF7L2: transcription factor 7 like 2
TGF-β: transforming growth-factor β
TiO_2_: titanium dioxide
Treg: regulatory T cell
TSA: trichostatin A
TXNIP: thioredoxin interacting protein
VEGF: vascular endothelial growth factor
VEGFR1: vascular endothelial growth factor receptor 1/2
